# Bis(*N*,*N*′,*N*′′-triisopropyl­guanidinium) fumarate–fumaric acid (1/1)

**DOI:** 10.1107/S1600536812023094

**Published:** 2012-05-26

**Authors:** Farouq F. Said, Basem F. Ali, Darrin Richeson, Ilia Korobkov

**Affiliations:** a Department of Chemistry, Al al-Bayt University, Mafraq 25113, Jordan; b Department of Chemistry and Biochemistry, University of Ottawa, Ottawa, Ontario, Canada K1N 6N5

## Abstract

The asymmetric unit of the title compound, C_10_H_24_N_3_^+^·0.5C_4_H_2_O_4_^2−^·0.5C_4_H_4_O_4_, comprises a triisopropyl­guanidinium cation, half of a fumarate dianion and half of a fumaric acid mol­ecule; both the fumarate dianion and the fumaric acid mol­ecule are located on inversion centres. In the crystal, inter­molecular O—H⋯O hydrogen bonds between the carboxyl groups of the fumaric acid mol­ecules and the carboxyl­ate groups of the fumarate anions lead to the formation of a hydrogen-bonded supra­molecular twisted chain along the *b* axis. The triisopropyl­guanidinium cations inter­act with the fumarate–fumaric acid chains *via* extensive N—H⋯O and C—H⋯O hydrogen bonds, leading to a ladder arrangement, with the cation being the rungs that bridge three curled chains of fumarate–fumaric acid. The crystal packing is stabilized by N—H⋯O and C—H⋯O (cation⋯fumarate/fumaric) and O—H⋯O (fumarate⋯fumaric) hydrogen bonds, consolidating a three-dimensional network.

## Related literature

For background information and *N*,*N′*,*N"*-tris­ubstituted guanidinium salts, see: Said *et al.* (2011 ▶). For related structures, see: Said *et al.* (2005 ▶); Hemamalini & Fun (2010 ▶); Büyükgüngör *et al.* (2004 ▶). For the preparation of the triisopropyl guanidine compound, see: Ong *et al.* (2003 ▶).
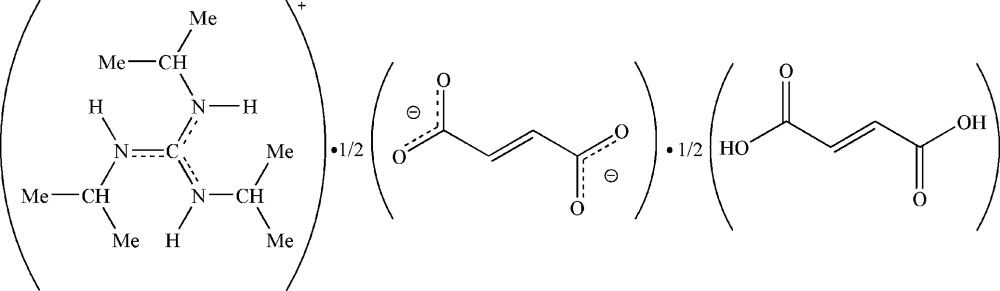


## Experimental

### 

#### Crystal data

C_10_H_24_N_3_^+^·0.5C_4_H_2_O_4_^2−^·0.5C_4_H_4_O_4_
*M* *_r_* = 301.39
Monoclinic, 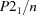

*a* = 9.714 (3) Å
*b* = 11.633 (3) Å
*c* = 16.226 (4) Å
β = 102.291 (4)°
*V* = 1791.6 (8) Å^3^
*Z* = 4
Mo *K*α radiation
μ = 0.08 mm^−1^
*T* = 200 K
0.50 × 0.45 × 0.45 mm

#### Data collection

Bruker APEXII CCD area-detector diffractometer
Absorption correction: multi-scan (*SADABS*; Bruker, 2003 ▶) *T*_min_ = 0.960, *T*_max_ = 0.964
11184 measured reflections
2514 independent reflections
2121 reflections with *I* > 2σ(*I*)
*R*_int_ = 0.027
θ_max_ = 23.3°

#### Refinement

*R*[*F* ^2^ > 2σ(*F* ^2^)] = 0.044
*wR*(*F* ^2^) = 0.130
*S* = 1.04
2514 reflections
191 parameters
H-atom parameters constrained
Δρ_max_ = 0.22 e Å^−3^
Δρ_min_ = −0.18 e Å^−3^

### 

Data collection: *APEX2* (Bruker, 2009 ▶); cell refinement: *SAINT* (Bruker, 2009 ▶); data reduction: *SAINT*; program(s) used to solve structure: *SHELXS97* (Sheldrick, 2008 ▶); program(s) used to refine structure: *SHELXL97* (Sheldrick, 2008 ▶); molecular graphics: *SHELXTL* (Sheldrick, 2008 ▶); software used to prepare material for publication: *SHELXTL*.

## Supplementary Material

Crystal structure: contains datablock(s) I, global. DOI: 10.1107/S1600536812023094/pv2540sup1.cif

Structure factors: contains datablock(s) I. DOI: 10.1107/S1600536812023094/pv2540Isup2.hkl

Additional supplementary materials:  crystallographic information; 3D view; checkCIF report

## Figures and Tables

**Table 1:** Hydrogen-bond geometry (Å, °)

*D*—H⋯*A*	*D*—H	H⋯*A*	*D*⋯*A*	*D*—H⋯*A*
N1—H1*A*⋯O3	0.88	2.05	2.866 (2)	154
N2—H2*A*⋯O4^i^	0.88	2.22	2.976 (2)	144
N3—H3*A*⋯O1^ii^	0.88	2.04	2.866 (2)	155
O2—H2⋯O4^iii^	0.84	1.66	2.484 (2)	168
C8—H8*A*⋯O3	1.00	2.49	3.356 (2)	144
C2—H2*B*⋯O4^i^	1.00	2.46	3.372 (2)	150
C5—H5*A*⋯O1^ii^	1.00	2.48	3.270 (2)	135

Symmetry codes: (i) 
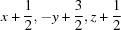
; (ii) 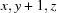; (iii) 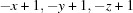.

**Fig. 1.**
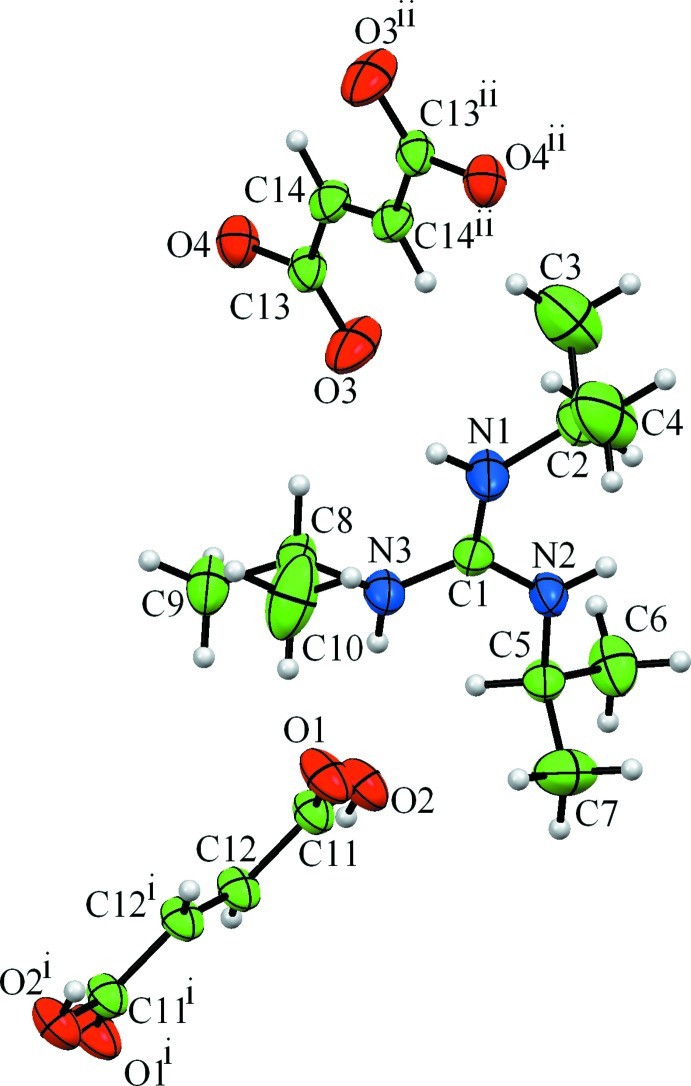
The molecular structure of the title compound with the atom numbering scheme. Displacement ellipsoids are drawn at the 50% probability level. H atoms are presented as small spheres of arbitrary radius. Symmetry operations: (i) 2 -x,-y, 1 -z; (ii) -x, 1 -y, 1 -z.

**Fig. 2.**
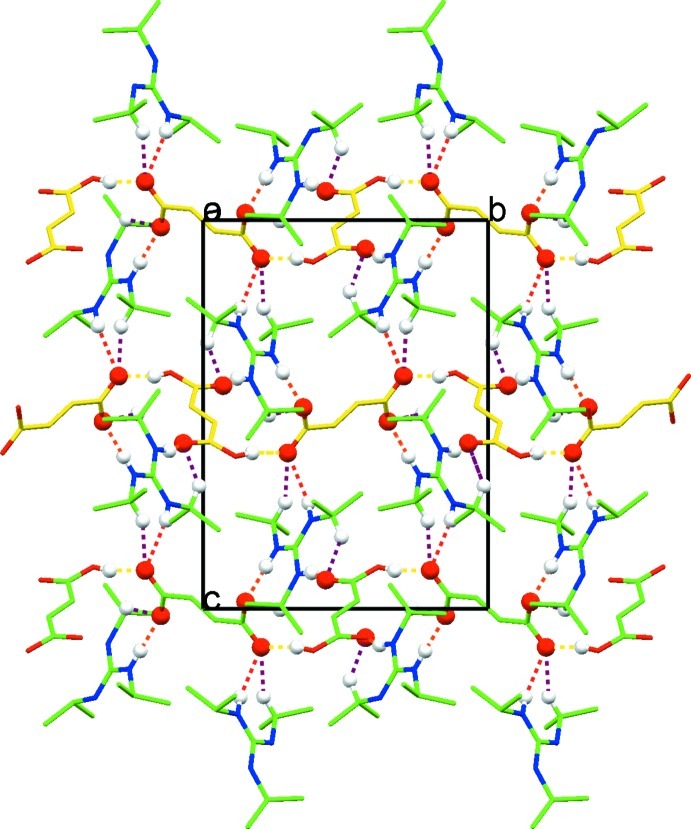
A view of the hydrogen bonding interactions (dotted lines) in the crystal structure of the title compound. H atoms non-participating in hydrogen-bonding were omitted for clarity.

## supplementary crystallographic information

### Comment

In connection with ongoing studies of the structural aspects of *N*,*N'*,*N"*-trisubstituted guanidinium salts (Said *et al.*, 2011), we herein report the crystal structure of the title compound (Fig. 1). The bond distances and bond angles in the title compound agree very well with the corresponding bond distances and bond angles reported in a similar compound earlier (Said *et al.*, 2005). The central guanidinium fragment of the cation is planar (sum of NCN angles is 360°). Both the fumarate and the fumaric acid units are planar and centrosymmetric with the inversion center at the midpoint of the C═C double bond. The C13—O3/O4 bonds in the fumarate dianion [1.222 (2) and 1.280 (2) Å] indicate a delocalized π-bonding arrangement as a consequence of deprotonation of the carboxylic acid group. On the other hand, the fumaric acid moiety displays a shorter C11—O1 bond [1.219 (3) Å] and a longer C11—O2 bond [1.302 (3) Å] as expected for a protonated carboxyl group. The carboxyl groups of the fumaric acid molecules and the carboxylate groups of the fumarate anions are hydrogen bonded through O2—H2···O4 leading to the formation of a one-dimensional hydrogen-bonded supramolecular twisting chain along the *b* -axis (Fig. 2, Table 1). This type of carboxyl-carboxylate interaction has been reported in the several crystal structures containing fumarate-fumaric acid species with different cations (Hemamalini & Fun, 2010, Büyükgüngör *et al.*, 2004) indicating the stability of such a supramolecular motif. The triisopropyl guanidinium cations are bridging three fumarate-fumaric curled chains *via* extensive N—H···O hydrogen bonds (Table 1), forming triply bridged twisted chains, leading to a ladder type arrangement with guanidinium cation forming rungs (Fig. 2). The extensive hydrogen bonding interactions between the fumarate-fumaric acid chains and the ladder of guanidinium rungs along the *b*-axis consolidate the three-dimensional network.

### Experimental

*N*,*N'*,*N"*-Triisopropylguanidine was prepared according to literature methods (Ong *et al.*, 2003). In a round bottom flask, a mixture fumaric acid (0.395 mmol) and *N*,*N'*,*N"*-triisopropylguanidine (0.395 mmol was dissolved in THF (10 ml). The reaction mixture was stirred, and a colorless precipitate formed over the next few minutes. The solid was removed by filtration and the product was crystallized from a mixture of THF:methanol (1:2) to give colorless crystals of the title compound (92% yield).

### Refinement

Hydrogen atoms were included in calculated positions and refined as riding on their parent atoms with N—H = 0.88 Å, O—H = 0.84 Å and C—H = 0.95–1.0 Å and *U*_iso_(H) = 1.2*U*_eq_(non-methyl C/N) or 1.5*U*_eq_(methyl C/O). Due to the quality of crystal we did not observe significant diffraction data past 0.95 Å resolution, therefore the data set was trimmed to that value to reduce data to noise ratio and improve the quality of the final refinement.

### Crystal data

**Table:**

C_10_H_24_N_3_^+^·0.5C_4_H_2_O_4_^2^^−^·0.5C_4_H_4_O_4_	*F*(000) = 656
*M**_r_* = 301.39	*D*_x_ = 1.117 Mg m^−^^3^*D*_m_ = n/a Mg m^−^^3^*D*_m_ measured by not measured
Monoclinic, *P*2_1_/*n*	Mo *K*α radiation, λ = 0.71073 Å
Hall symbol: -P 2yn	Cell parameters from 309 reflections
*a* = 9.714 (3) Å	θ = 2.2–23.3°
*b* = 11.633 (3) Å	µ = 0.08 mm^−^^1^
*c* = 16.226 (4) Å	*T* = 200 K
β = 102.291 (4)°	Block, colourless
*V* = 1791.6 (8) Å^3^	0.50 × 0.45 × 0.45 mm
*Z* = 4	

### Data collection

**Table:**

Bruker APEXII CCD area-detector diffractometer	2514 independent reflections
Radiation source: fine-focus sealed tube	2121 reflections with *I* > 2σ(*I*)
Graphite monochromator	*R*_int_ = 0.027
phi and ω scans	θ_max_ = 23.3°, θ_min_ = 2.2°
Absorption correction: multi-scan (*SADABS*; Bruker, 2003)	*h* = −10→10
*T*_min_ = 0.960, *T*_max_ = 0.964	*k* = −12→12
11184 measured reflections	*l* = −18→18

### Refinement

**Table:**

Refinement on *F*^2^	Secondary atom site location: difference Fourier map
Least-squares matrix: full	Hydrogen site location: inferred from neighbouring sites
*R*[*F*^2^ > 2σ(*F*^2^)] = 0.044	H-atom parameters constrained
*wR*(*F*^2^) = 0.130	*w* = 1/[σ^2^(*F*_o_^2^) + (0.0727*P*)^2^ + 0.7515*P*] where *P* = (*F*_o_^2^ + 2*F*_c_^2^)/3
*S* = 1.04	(Δ/σ)_max_ = 0.001
2514 reflections	Δρ_max_ = 0.22 e Å^−^^3^
191 parameters	Δρ_min_ = −0.18 e Å^−^^3^
0 restraints	Extinction correction: *SHELXL97* (Sheldrick, 2008), Fc^*^=kFc[1+0.001xFc^2^λ^3^/sin(2θ)]^-1/4^
Primary atom site location: structure-invariant direct methods	Extinction coefficient: 0.013 (2)

### Special details

**Table:**

Experimental. Data collection is performed with three batch runs at phi = 0.00 ° (650 frames), at phi = 120.00 ° (650 frames), and at phi = 240.00 ° (650 frames). Frame width = 0.30 ° in omega. Data is merged, corrected for decay (if any), and treated with multi-scan absorption corrections (if required). All symmetry-equivalent reflections are merged for centrosymmetric data. Friedel pairs are not merged for noncentrosymmetric data.
Geometry. All e.s.d.'s (except the e.s.d. in the dihedral angle between two l.s. planes) are estimated using the full covariance matrix. The cell e.s.d.'s are taken into account individually in the estimation of e.s.d.'s in distances, angles and torsion angles; correlations between e.s.d.'s in cell parameters are only used when they are defined by crystal symmetry. An approximate (isotropic) treatment of cell e.s.d.'s is used for estimating e.s.d.'s involving l.s. planes.
Refinement. Refinement of *F*^2^ against ALL reflections. The weighted *R*-factor *wR* and goodness of fit *S* are based on *F*^2^, conventional *R*-factors *R* are based on *F*, with *F* set to zero for negative *F*^2^. The threshold expression of *F*^2^ > σ(*F*^2^) is used only for calculating *R*-factors(gt) *etc*. and is not relevant to the choice of reflections for refinement. *R*-factors based on *F*^2^ are statistically about twice as large as those based on *F*, and *R*- factors based on ALL data will be even larger.

### Fractional atomic coordinates and isotropic or equivalent isotropic displacement parameters (Å^2^)

**Table:**

	*x*	*y*	*z*	*U*_iso_*/*U*_eq_	
N1	0.3813 (2)	0.7589 (2)	0.65518 (12)	0.0606 (6)	
H1A	0.3496	0.7206	0.6082	0.073*	
N2	0.55340 (17)	0.87868 (16)	0.72883 (10)	0.0407 (5)	
H2A	0.5134	0.8698	0.7722	0.049*	
N3	0.55035 (18)	0.83370 (15)	0.58961 (10)	0.0400 (5)	
H3A	0.6221	0.8809	0.5930	0.048*	
C1	0.4961 (2)	0.82257 (18)	0.65809 (12)	0.0372 (5)	
C2	0.3022 (3)	0.7462 (3)	0.72241 (15)	0.0618 (8)	
H2B	0.3531	0.7879	0.7738	0.074*	
C3	0.1559 (4)	0.7963 (3)	0.6947 (2)	0.0908 (10)	
H3B	0.1630	0.8780	0.6813	0.136*	
H3C	0.1047	0.7880	0.7402	0.136*	
H3D	0.1053	0.7554	0.6445	0.136*	
C4	0.2946 (4)	0.6209 (3)	0.7427 (2)	0.0980 (12)	
H4A	0.3902	0.5903	0.7614	0.147*	
H4B	0.2463	0.5794	0.6922	0.147*	
H4C	0.2424	0.6111	0.7876	0.147*	
C5	0.6772 (2)	0.95385 (17)	0.74056 (13)	0.0377 (5)	
H5A	0.6841	0.9853	0.6842	0.045*	
C6	0.6565 (3)	1.0533 (2)	0.79649 (16)	0.0575 (7)	
H6A	0.5696	1.0942	0.7712	0.086*	
H6B	0.7367	1.1061	0.8023	0.086*	
H6C	0.6500	1.0243	0.8522	0.086*	
C7	0.8100 (3)	0.8884 (2)	0.77559 (19)	0.0652 (7)	
H7A	0.8199	0.8251	0.7374	0.098*	
H7B	0.8056	0.8574	0.8311	0.098*	
H7C	0.8911	0.9401	0.7811	0.098*	
C8	0.5007 (2)	0.77405 (19)	0.50895 (13)	0.0438 (6)	
H8A	0.3956	0.7697	0.4980	0.053*	
C9	0.5406 (3)	0.8431 (2)	0.43942 (14)	0.0555 (6)	
H9A	0.5003	0.9204	0.4387	0.083*	
H9B	0.5041	0.8054	0.3852	0.083*	
H9C	0.6434	0.8486	0.4490	0.083*	
C10	0.5571 (4)	0.6538 (2)	0.51258 (18)	0.0841 (10)	
H10A	0.5288	0.6120	0.5587	0.126*	
H10B	0.6602	0.6561	0.5223	0.126*	
H10C	0.5193	0.6147	0.4591	0.126*	
C11	0.8673 (2)	0.02478 (18)	0.57016 (13)	0.0372 (5)	
C12	0.9785 (2)	0.04210 (18)	0.52111 (13)	0.0366 (5)	
H12A	1.0196	0.1161	0.5202	0.044*	
C13	0.0836 (2)	0.63781 (18)	0.46512 (12)	0.0373 (5)	
C14	−0.0139 (2)	0.54111 (18)	0.47181 (12)	0.0388 (5)	
H14A	−0.1018	0.5388	0.4327	0.047*	
O1	0.81517 (17)	−0.06979 (13)	0.57428 (11)	0.0534 (5)	
O2	0.82763 (15)	0.11397 (12)	0.60762 (10)	0.0468 (4)	
H2	0.8741	0.1718	0.5986	0.070*	
O3	0.19305 (19)	0.64871 (16)	0.51791 (11)	0.0672 (6)	
O4	0.04379 (15)	0.70345 (12)	0.40121 (9)	0.0425 (4)	

### Atomic displacement parameters (Å^2^)

**Table:**

	*U*^11^	*U*^22^	*U*^33^	*U*^12^	*U*^13^	*U*^23^
N1	0.0536 (12)	0.0929 (16)	0.0369 (10)	−0.0418 (11)	0.0131 (9)	−0.0110 (10)
N2	0.0404 (10)	0.0532 (11)	0.0306 (9)	−0.0154 (8)	0.0123 (7)	−0.0032 (8)
N3	0.0414 (10)	0.0462 (10)	0.0337 (9)	−0.0158 (8)	0.0111 (7)	−0.0042 (8)
C1	0.0352 (11)	0.0435 (12)	0.0321 (11)	−0.0100 (9)	0.0058 (9)	0.0015 (9)
C2	0.0527 (14)	0.094 (2)	0.0402 (13)	−0.0384 (14)	0.0138 (11)	−0.0032 (13)
C3	0.093 (2)	0.087 (2)	0.106 (3)	0.0130 (18)	0.051 (2)	0.0107 (19)
C4	0.091 (2)	0.116 (3)	0.098 (2)	0.018 (2)	0.0448 (19)	0.059 (2)
C5	0.0373 (11)	0.0419 (12)	0.0344 (11)	−0.0107 (9)	0.0085 (9)	−0.0029 (9)
C6	0.0753 (17)	0.0488 (15)	0.0524 (14)	−0.0164 (12)	0.0227 (13)	−0.0100 (11)
C7	0.0413 (13)	0.0687 (17)	0.0832 (19)	−0.0008 (12)	0.0076 (13)	0.0107 (14)
C8	0.0505 (13)	0.0483 (13)	0.0303 (11)	−0.0115 (10)	0.0035 (9)	−0.0018 (9)
C9	0.0729 (16)	0.0591 (15)	0.0346 (12)	−0.0105 (12)	0.0115 (11)	−0.0006 (11)
C10	0.143 (3)	0.0518 (17)	0.0493 (16)	0.0038 (17)	0.0019 (17)	−0.0053 (12)
C11	0.0385 (11)	0.0384 (12)	0.0380 (11)	−0.0092 (9)	0.0154 (9)	−0.0038 (9)
C12	0.0381 (11)	0.0344 (11)	0.0404 (11)	−0.0115 (8)	0.0152 (9)	−0.0012 (8)
C13	0.0428 (12)	0.0405 (12)	0.0291 (11)	−0.0111 (9)	0.0089 (9)	−0.0064 (9)
C14	0.0378 (11)	0.0453 (12)	0.0313 (10)	−0.0120 (9)	0.0028 (9)	−0.0033 (8)
O1	0.0598 (10)	0.0442 (9)	0.0669 (11)	−0.0215 (8)	0.0376 (8)	−0.0112 (8)
O2	0.0534 (9)	0.0382 (8)	0.0578 (9)	−0.0073 (7)	0.0316 (8)	−0.0029 (7)
O3	0.0652 (11)	0.0750 (12)	0.0514 (10)	−0.0411 (9)	−0.0100 (9)	0.0121 (9)
O4	0.0539 (9)	0.0377 (8)	0.0366 (8)	−0.0069 (7)	0.0111 (7)	0.0000 (6)

### Geometric parameters (Å, º)

**Table:**

N1—C1	1.331 (3)	C7—H7A	0.9800
N1—C2	1.469 (3)	C7—H7B	0.9800
N1—H1A	0.8800	C7—H7C	0.9800
N2—C1	1.335 (3)	C8—C10	1.499 (4)
N2—C5	1.466 (3)	C8—C9	1.502 (3)
N2—H2A	0.8800	C8—H8A	1.0000
N3—C1	1.334 (3)	C9—H9A	0.9800
N3—C8	1.469 (3)	C9—H9B	0.9800
N3—H3A	0.8800	C9—H9C	0.9800
C2—C4	1.500 (5)	C10—H10A	0.9800
C2—C3	1.513 (4)	C10—H10B	0.9800
C2—H2B	1.0000	C10—H10C	0.9800
C3—H3B	0.9800	C11—O1	1.219 (2)
C3—H3C	0.9800	C11—O2	1.302 (3)
C3—H3D	0.9800	C11—C12	1.485 (3)
C4—H4A	0.9800	C12—C12^i^	1.314 (4)
C4—H4B	0.9800	C12—H12A	0.9500
C4—H4C	0.9800	C13—O3	1.222 (3)
C5—C7	1.501 (3)	C13—O4	1.279 (3)
C5—C6	1.510 (3)	C13—C14	1.489 (3)
C5—H5A	1.0000	C14—C14^ii^	1.311 (4)
C6—H6A	0.9800	C14—H14A	0.9500
C6—H6B	0.9800	O2—H2	0.8400
C6—H6C	0.9800		
			
C1—N1—C2	126.59 (19)	H6A—C6—H6C	109.5
C1—N1—H1A	116.7	H6B—C6—H6C	109.5
C2—N1—H1A	116.7	C5—C7—H7A	109.5
C1—N2—C5	125.70 (16)	C5—C7—H7B	109.5
C1—N2—H2A	117.1	H7A—C7—H7B	109.5
C5—N2—H2A	117.1	C5—C7—H7C	109.5
C1—N3—C8	125.69 (17)	H7A—C7—H7C	109.5
C1—N3—H3A	117.2	H7B—C7—H7C	109.5
C8—N3—H3A	117.2	N3—C8—C10	110.97 (19)
N1—C1—N2	119.70 (18)	N3—C8—C9	109.17 (18)
N1—C1—N3	120.07 (18)	C10—C8—C9	112.2 (2)
N2—C1—N3	120.18 (17)	N3—C8—H8A	108.1
N1—C2—C4	108.6 (2)	C10—C8—H8A	108.1
N1—C2—C3	110.3 (2)	C9—C8—H8A	108.1
C4—C2—C3	110.6 (2)	C8—C9—H9A	109.5
N1—C2—H2B	109.1	C8—C9—H9B	109.5
C4—C2—H2B	109.1	H9A—C9—H9B	109.5
C3—C2—H2B	109.1	C8—C9—H9C	109.5
C2—C3—H3B	109.5	H9A—C9—H9C	109.5
C2—C3—H3C	109.5	H9B—C9—H9C	109.5
H3B—C3—H3C	109.5	C8—C10—H10A	109.5
C2—C3—H3D	109.5	C8—C10—H10B	109.5
H3B—C3—H3D	109.5	H10A—C10—H10B	109.5
H3C—C3—H3D	109.5	C8—C10—H10C	109.5
C2—C4—H4A	109.5	H10A—C10—H10C	109.5
C2—C4—H4B	109.5	H10B—C10—H10C	109.5
H4A—C4—H4B	109.5	O1—C11—O2	121.74 (18)
C2—C4—H4C	109.5	O1—C11—C12	120.65 (18)
H4A—C4—H4C	109.5	O2—C11—C12	117.61 (17)
H4B—C4—H4C	109.5	C12^i^—C12—C11	121.8 (2)
N2—C5—C7	111.20 (19)	C12^i^—C12—H12A	119.1
N2—C5—C6	108.94 (17)	C11—C12—H12A	119.1
C7—C5—C6	112.0 (2)	O3—C13—O4	125.02 (19)
N2—C5—H5A	108.2	O3—C13—C14	119.94 (19)
C7—C5—H5A	108.2	O4—C13—C14	115.03 (18)
C6—C5—H5A	108.2	C14^ii^—C14—C13	124.2 (2)
C5—C6—H6A	109.5	C14^ii^—C14—H14A	117.9
C5—C6—H6B	109.5	C13—C14—H14A	117.9
H6A—C6—H6B	109.5	C11—O2—H2	109.5
C5—C6—H6C	109.5		
			
C2—N1—C1—N2	3.1 (4)	C1—N2—C5—C7	−92.6 (3)
C2—N1—C1—N3	−174.4 (2)	C1—N2—C5—C6	143.5 (2)
C5—N2—C1—N1	−178.4 (2)	C1—N3—C8—C10	−80.4 (3)
C5—N2—C1—N3	−1.0 (3)	C1—N3—C8—C9	155.4 (2)
C8—N3—C1—N1	−4.8 (3)	O1—C11—C12—C12^i^	1.9 (4)
C8—N3—C1—N2	177.8 (2)	O2—C11—C12—C12^i^	−177.6 (3)
C1—N1—C2—C4	−123.8 (3)	O3—C13—C14—C14^ii^	−5.4 (4)
C1—N1—C2—C3	114.8 (3)	O4—C13—C14—C14^ii^	173.6 (3)

Symmetry codes: (i) −*x*+2, −*y*, −*z*+1; (ii) −*x*, −*y*+1, −*z*+1.

### Hydrogen-bond geometry (Å, º)

**Table:**

*D*—H···*A*	*D*—H	H···*A*	*D*···*A*	*D*—H···*A*
N1—H1*A*···O3	0.88	2.05	2.866 (2)	154
N2—H2*A*···O4^iii^	0.88	2.22	2.976 (2)	144
N3—H3*A*···O1^iv^	0.88	2.04	2.866 (2)	155
O2—H2···O4^v^	0.84	1.66	2.484 (2)	168
C8—H8*A*···O3	1.00	2.49	3.356 (2)	144
C2—H2*B*···O4^iii^	1.00	2.46	3.372 (2)	150
C5—H5*A*···O1^iv^	1.00	2.48	3.270 (2)	135

Symmetry codes: (iii) *x*+1/2, −*y*+3/2, *z*+1/2; (iv) *x*, *y*+1, *z*; (v) −*x*+1, −*y*+1, −*z*+1.
